# Left ventricular blood cyst in an adult case

**DOI:** 10.1002/ccr3.1083

**Published:** 2017-07-20

**Authors:** Hirohiko Akutsu, Arata Muraoka, Yoshio Misawa

**Affiliations:** ^1^ Division of Cardiovascular Surgery Department of Surgery Jichi Medical University Shimotsuke Japan

**Keywords:** Cardiac blood cyst, cardiac tumor, echocardiography, mitral regurgitation

## Abstract

We present a 57‐year‐old man with a 1.5‐cm‐diameter blood cyst in the left ventricle, which was incidentally detected by conventional diagnostic echocardiography before colon surgery. The cyst originated from the papillary muscle, and the pathological findings were compatible with a cardiac blood cyst.

## Introduction

Most cardiac tumors are benign and incidentally detected. Blood cysts are rare benign lesions. Small blood cysts in the cardiac chamber are sometimes recognized during operations in infants, and they generally show no clinical adverse effects because of their small size. Cardiac blood cysts are very rarely detected preoperatively.

## Case Presentation

**Figure 1:**
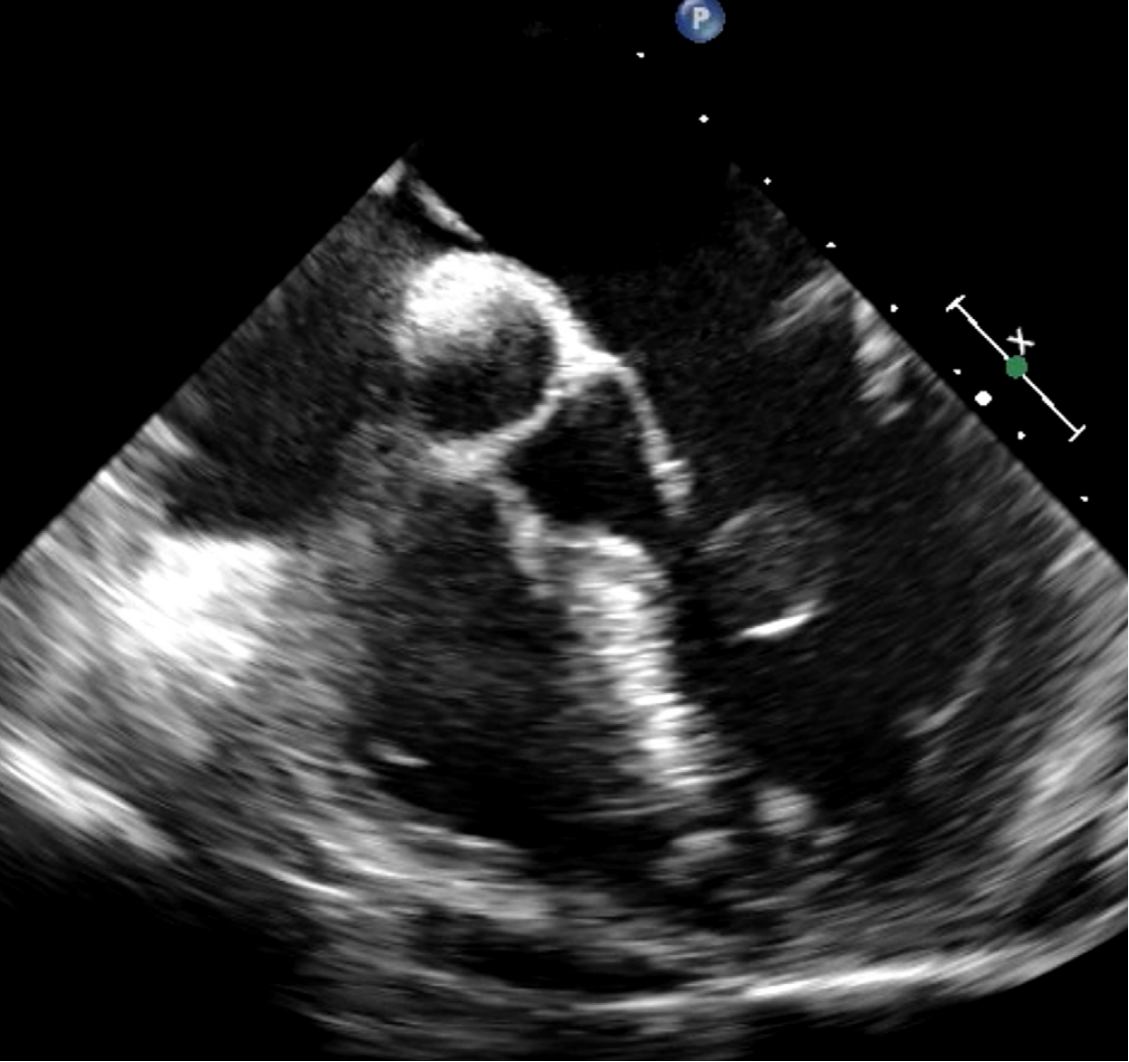
Preoperative transesophageal echocardiography. A 15‐mm cystic round mass was observed in the left ventricle.

A 57‐year‐old man was transferred to our hospital for treatment of an asymptomatic cardiac tumor. The tumor was incidentally detected during a preoperative work‐up for colon cancer. On examination, he was afebrile with no cardiac symptoms. A Levine II/IV pansystolic murmur was audible at the apex area without an arrhythmia. The patient's blood cell counts were normal, and his C‐reactive protein concentration was <0.1 mg/mL with normal renal and liver function. Chest radiographs showed no cardiomegaly and clear lung fields. Echocardiography revealed a 1.5‐cm‐diameter cystic mass in the left ventricle with moderate mitral valve regurgitation, and transesophageal echocardiography indicated that the tumor seemed to be attached the mitral chorda of the anterior leaflet (Fig. 1).

**Figure 2:**
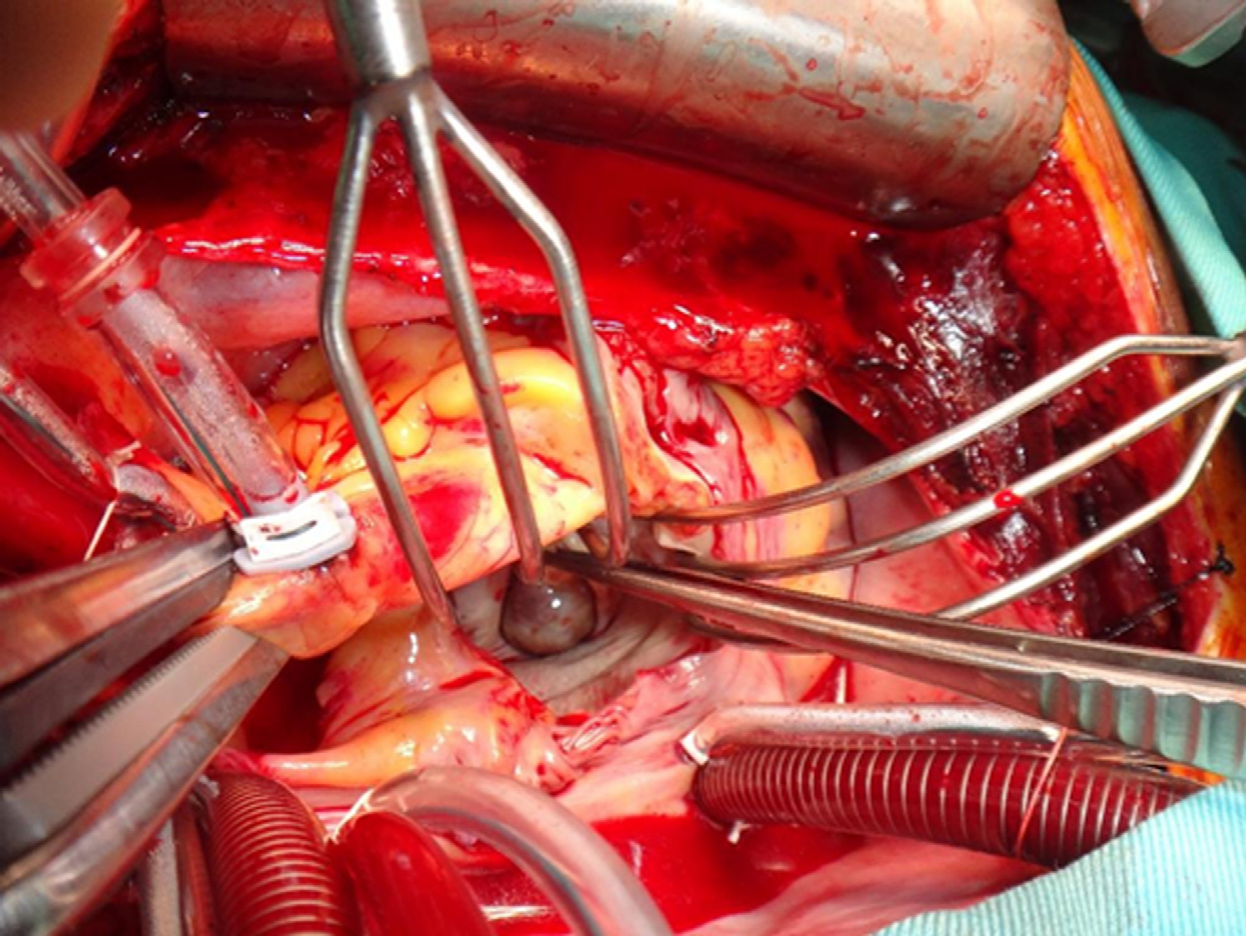
Intraoperative findings. The surface of the mass was round and smooth. The mass was attached to the rough zone of the anterior leaflet of the mitral valve.

We could not rule out the malignant potential of the tumor and its possible risks of embolic stroke. We chose a superior septal approach to obtain an excellent operative view within the left ventricle. The tumor was attached to the rough zone of the anterior leaflet and was free from the chordaetendineae. A traction suture was placed in the tumor, and bloody fluid ran from the tumor, which finally collapsed (Fig. 2). The tumor originated from the papillary muscle and was resected with a small amount of rough zone. Artificial chordaetendineae were used to repair the mitral valve regurgitation associated with partial ring annuloplasty.

**Figure 3:**
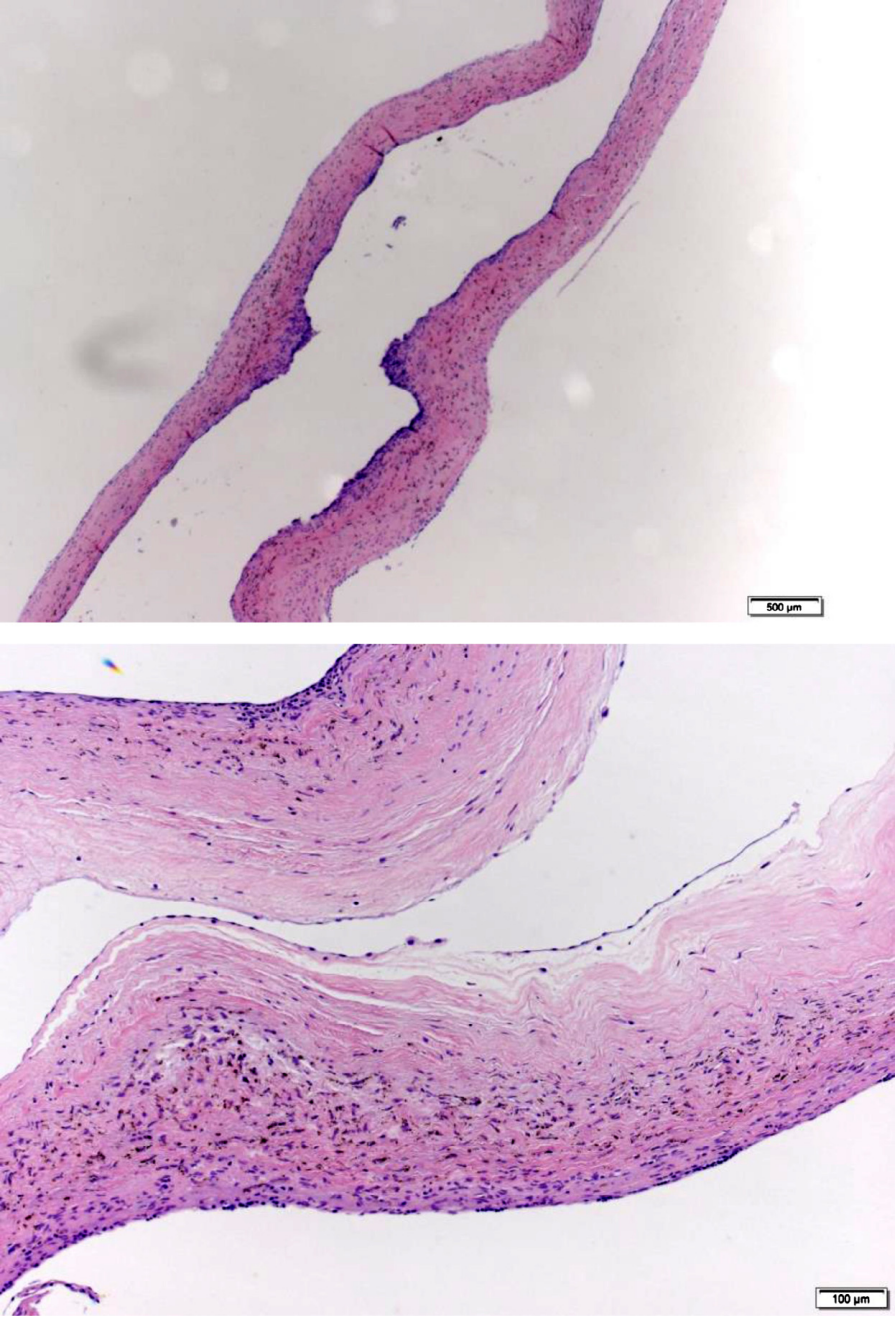
Pathological finding of the resected mass. A single layer of endothelium lined the surface of the mass. No blood clot was found in the mass.

The pathological findings were compatible with a cardiac blood cyst (Fig. 3). The tumor wall comprised thin‐layered fibrous tissue lined with a single layer of endothelium, and no tumor cells were found. Postoperative echocardiography revealed a small amount of mitral regurgitation. The postoperative course was uneventful, and good left ventricular and mitral valve functions were confirmed.

## Discussion

Cardiac blood cysts might spontaneously regress or rarely grow to sizes detectable by conventional prognostic procedures. Cardiac cysts may develop in any heart cavity.

Okubo et al. 1 described a 6‐year‐old girl with a 30‐mm round cyst in the right atrium, and Mori et al. 2 described a 69‐year‐old man with a 25‐ × 22‐mm mass in the right atrium. Ohmoto et al. 3 resected a mobile mass (30 × 20 × 40 mm) attached to the anterolateral papillary muscle in a 57‐year‐old man. All three of these patients had no symptoms, but their tumors were resected because of the possible risk of embolic stroke. Tsutsui et al. 4 diagnosed a blood cyst measuring 15 × 8 mm in the left ventricle with echocardiography and computed tomography and followed the patient, who was maintained on medical treatment of aspirin. Jacob et al. 5 reported a case involving a patient with embolic stroke and multiple blood cysts in the left ventricle. The authors presumed that the cyst might have caused the stroke, and they maintained anticoagulant therapy for the patient.

In general, microscopic findings of a blood cyst are consisted of fibrous tissue covered with normal endothelium. No tumorous proliferative cells are found. The findings of our case are consistent with previously reported cases 1, 3.

The optimal management of asymptomatic cysts has not yet reached consensus. Some clinicians routinely remove such cysts to exclude malignancy and avoid the potential risk of embolism, and others refrain from surgical resection of cystic masses that do not interfere with normal cardiac function.

## Conclusions

We have herein presented a case involving a 57‐year‐old man with an incidentally detected blood cyst in the left ventricle. The tumor originated from the papillary muscle and was attached to the rough zone of the anterior leaflet; it was successfully resected with a small amount of rough zone followed by repair of mitral valve regurgitation.

## Conflict of Interest

None declared.

## Authorship

HA and AM: participated in the design this study and helped competing an initial manuscript. YM: carried out its final manuscript. All authors read and approved the manuscript.
